# Barberry plays an active role as an alternate host of *Puccinia graminis* in Spain

**DOI:** 10.1111/ppa.13540

**Published:** 2022-03-09

**Authors:** Dolors Villegas, Radhika Bartaula, Carlos Cantero‐Martínez, Douglas Luster, Les Szabo, Pablo Olivera, Anna Berlin, Julian Rodriguez‐Algaba, Mogens S. Hovmøller, Robert McIntosh, Yue Jin

**Affiliations:** ^1^ IRTA Institute of Agrifood Research and Technology Lleida Spain; ^2^ Department of Plant Pathology University of Minnesota St Paul MN USA; ^3^ Universitat de Lleida Agrotecnio Center Lleida Spain; ^4^ USDA‐ARS Foreign Disease‐Weed Science Research Unit Ft Detrick MD USA; ^5^ USDA‐ARS Cereal Disease Laboratory University of Minnesota St Paul MN USA; ^6^ Department of Forest Mycology and Plant Pathology Swedish University of Agricultural Sciences Uppsala Sweden; ^7^ Department of Agroecology Global Rust Reference Center Aarhus University Slagelse Denmark; ^8^ University of Sydney Plant Breeding Institute School of Life and Environmental Sciences Cobbitty New South Wales Australia

**Keywords:** aecial host, *Berberis garciae*, *Berberis hispanica*, sexual cycle, stem rust

## Abstract

Stem rust, caused by *Puccinia graminis*, is a destructive group of diseases. The pathogen uses *Berberis* species as alternate hosts to complete its life cycle. *B*. *vulgaris* and the endemic species *B. hispanica* and *B. garciae* are present in Spain. The objective of this study was to investigate the functionality of the indigenous barberry as alternate hosts. Field surveys were conducted in 2018 and 2019 in Huesca, Teruel and Albacete provinces of Spain. Aecial samples on barberry were analysed via infection assays and DNA analysis. *B*. *garciae* was predominant in Huesca and Teruel provinces, often found in the field margins of cereal crops. Aecial infections on *B*. *garciae* were observed in May and uredinial infections on cereal crops in June. Scattered *B*. *hispanica* bushes were occasionally found near cereal crops in Albacete, where aecial infections on *B*. *hispanica* were observed in June when most cereal crops were mature. Infection assays using aeciospores resulted in stem rust infections on susceptible genotypes of wheat, barley, rye and oat, indicating the presence of the sexual cycle for *P*. *graminis* f. sp. *tritici*, f. sp. *secalis* and f. sp. *avenae*. Sequence analyses from aecial samples supported this finding as well as the presence of *Puccinia brachypodii*. This study provides the first evidence that indigenous *Berberis* species play an active role in the sexual cycle of *P*. *graminis* under natural conditions in Spain.

## Graphical abstract

Plants of indigenous *Berberis* spp. were frequently found growing near cereal crop fields in some areas of Spain and aeciospores from these barberries infected wheat, barley, rye and oats.

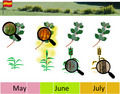

## 1 INTRODUCTION

Stem rust of cereal crops and grasses, caused by *Puccinia graminis*, is a destructive group of diseases that has caused human misery for more than a millennium (Kislev, 1982). *P. graminis* comprises specialized forms (or *formae speciales*, ff. sp.) that attack specific cereal crop and grass species, such as the form *P*. *graminis* f. sp. *tritici* (Pgt) that mainly attacks wheat. Barberry (*Berberis* spp.) serves as the aecial host where the sexual cycle of the fungus is completed. Many *Berberis* spp. are known to be susceptible to *P*. *graminis* (Levine & Cotter, 1932). Breeding stem rust‐resistant wheat varieties and large‐scale removal of common barberry (*Berberis vulgaris*) from major wheat‐growing regions resulted in effective control of the wheat stem rust in North America and western Europe during the first two thirds of the twentieth century (Peterson et al., 2005). However, over the last two decades some countries have experienced a resurgence of stem rust after many decades of quiescence (Berlin et al., 2015; Olivera Firpo et al., 2017; Olivera et al., 2015, 2019; Saunders et al., 2019). Part of this resurgence was caused by the evolution and continuing spread of *Sr31*‐virulent races, commonly known as the Ug99 race group, which began in East Africa in 1998 and radiated to adjacent countries and beyond over ensuing years. In 2013, multiple Pgt races with novel virulence combinations were detected from samples collected in central Germany where wheat stem rust outbreaks occurred (Olivera Firpo et al., 2017). In 2016, a highly virulent race, TTRTF, detected first in a sexual population from Georgia (Olivera et al., 2019), caused a severe outbreak in durum wheat in Sicily, Italy (Bhattacharya, 2017). Stem rust infections on cereals were observed in 10 European countries in recent years (Hovmøller et al., 2020), including the United Kingdom (Lewis et al., 2018), Denmark and Sweden (authors’ unpublished data).

New genetic variants of *P*. *graminis*, largely exampled by f. sp. *tritici*, can arise by both asexual and sexual mechanisms. Asexual variation independent of the presence of alternate hosts is caused by mutation of single avirulence factors or somatic recombination that is not well understood and more complex than simple nuclear exchange (Park & Wellings, 2012). The role of sexual recombination in generating genetic variability in *P*. *graminis* has been well documented (Craigie, 1927; Roelfs, 1982; Stakman et al., 1930). According to Roelfs (1982) and Peterson et al. (2005), the massive eradication of barberry in the United States from 1918 to 1974 had a large impact on reducing genetic variability and increasing the stability of Pgt races found in the US Great Plains. This topic has regained attention recently. The resurgence of stem rust in Europe after decades of near absence turned attention to barberry, with localized epidemics and identification of multiple pathogen races associated with the presence of barberry (Berlin et al., 2013; Olivera Firpo et al., 2017; Olivera et al., 2019; Saunders et al., 2019). Multiple races of Pgt with highly diverse virulence combinations were recovered from the Caucasus region (Olivera et al., 2019) and central Asia (Berlin et al., 2015). This implied that sexual recombination of Pgt on barberry is also common in these regions. Sexual recombination has two genetic aspects apart from the role of telia in cross‐season survival of the pathogen. First, recessive alleles for virulence carried by heterozygous avirulent individuals can become homozygous virulent; and secondly it generates new gene combinations (i.e., races), some of which might overcome deployed resistance gene combinations and therefore will be of a selective advantage.

Most of our knowledge on the role of barberry in wheat stem rust pathogen variation and disease epidemiology relates to common barberry, *B*. *vulgaris*. However, there is a wide range of *Berberis* spp. (and interspecific hybrids) and knowledge on their reaction to Pgt and association with cereal crops is limited. Although evidence is lacking, it is reasonably expected that there is genetic variation within barberry species and populations in regard to infection by *P*. *graminis*. Finally, *Berberis* spp. are alternate hosts for different graminaceous rust pathogens and the presence of pycnia or aecia on barberry plants is not sufficient to conclude a connection between cereal stem rust and barberry. Because the morphologies at the aecial stage of different *Puccinia* spp. infecting barberry are very similar, infection assays via inoculation experiments or DNA‐based diagnosis are needed to identify specific rust species in an aecial sample.

Spain is one of the major wheat‐producing countries in western Europe. Wheat occupied nearly 2 million hectares in 2018, 82% bread wheat and 18% durum wheat, with total production of 8 million tonnes (MAGRAMA, 2020). Wheat stem rust has not been considered a major problem in Spain in recent decades. A significant factor was the widespread adoption of early maturing stem rust‐resistant varieties in the 1960s, after which reports of stem rust became quite sporadic (Martínez‐Moreno & Solis, 2019).

**FIGURE 1:**
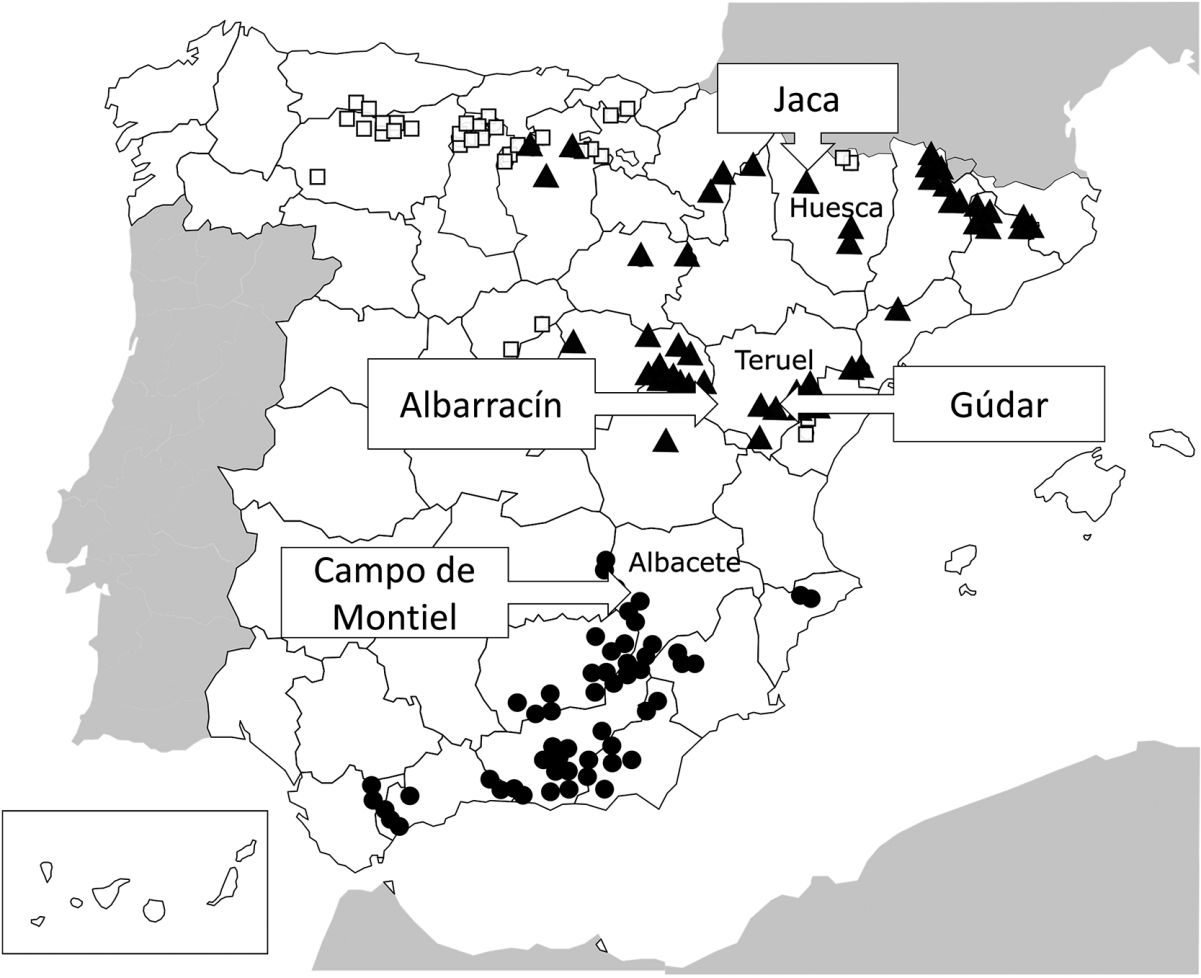
Geographical distribution of *Berberis* spp. in Spain and locations where surveys were conducted in 2018 and 2019. White squares indicate locations of *B*. *vulgaris*, black triangles indicate *B*. *garciae* and black circles indicate *B*. *hispanica*. Text boxes indicate the locations where surveys were carried out. Modified from the Anthos website (http://www.anthos.es)

Several *Berberis* spp. are present in Spain (Lopez González, 1986; Figure 1). *Berberis hispanica* (syn. *B*. *vulgaris* subsp. *australis*) is endemic to the southern Iberia Peninsula (Albacete, Almería, Cádiz, Ciudad Real, Granada, Jaén, Málaga and Murcia provinces) as well as in the Atlas Mountains in Morocco and Algeria. *B*. *garciae* (syn. *B*. *vulgaris* subsp. *seroi*) is endemic to the north‐eastern part of the country. *B*. *vulgaris* (syn. *B*. *vulgaris* subsp. *vulgaris*) is presumably a naturalized species in Spain and is mainly distributed in the northern part of the country, occasionally codistributed with *B*. *garciae* in the north‐east. Forms intermediate between *B*. *garciae* and *B*. *vulgaris*, probably of interspecific hybrids, are also present in the north‐eastern part of the country (Monserrat, 1970).

The rust pathogen flora on *Berberis* spp. in the Iberian Peninsula is unknown, and the responses of *B*. *hispanica* and *B*. *garciae* to *P*. *graminis* infection have not been investigated. Earlier observations indicated that the alternate host probably played an active role in stem rust epidemiology and pathogen variation more than half a century ago. Visiting wheat fields in Spain in the 1920s, Stakman (1923) observed stem rust infections only in fields near barberry bushes. Urríes and Cañamas (1952) recovered a large and diverse group of Pgt races from aecial samples collected from *B*. *hispanica* in 1950 and 1951. This contrasted with a limited number of races identified from uredinial samples collected from wheat during the same years. Salazar and Brañas (1973) also reported that races recovered from rusted wheat samples collected near barberry bushes were different from races identified from samples collected in fields without adjacent barberry. The unusual virulence combination (including virulence for *Sr31*) of a Pgt isolate derived from a wheat stem sample collected near barberry in Monteagudo del Castillo (Teruel province, Spain) further implicated the sexual cycle in barberry (Olivera et al., 2022). The objective of the present study was to investigate the functionality of indigenous *Berberis* spp. in Spain as the alternate hosts for *P*. *graminis*.

## 2 MATERIALS AND METHODS

### 2.1 Field surveys

Field surveys were conducted in 2018 and 2019 in Huesca, Teruel and Albacete provinces, Spain, to collect aecial samples on barberry and record the presence of uredinia on cereal crops and grasses that might serve as accessory hosts (Figure 1). The sites for more detailed survey were chosen based on a preliminary survey to identify locations of indigenous *Berberis* spp. in 2015 and *Berberis* distribution reported in the botanical database Anthos (http://www.anthos.es; Figure 1). Ten sites (Table 1) were surveyed over 6 months between April and October, when barberry plants and grasses are infected with various stages of rust fungi. Table S1 shows the sampling calendar and observations of infections for 2019.

**TABLE 1:** Characteristics of surveyed sites: location, site, barberry species sampled, main cereal crops covering the surveyed area and predominant grasses

Location, province	Site	*Berberis* species sampled	Main cereal crop species^a^	Predominant grasses	Predominant grasses with stem rust infections
Jaca, Huesca	Larrés	*B. garciae*	Wheat (36%), barley (38%), triticale (19%), rye (4%), oats (2%)	*Dactylis glomerata*, *Elymus repens*, *Brachypodium phoenicoides*	*D*. *glomerata*, *E*. *repens*
	Hostal de Ipies	Hybrid (*B*. *garciae* × *B*. *vulgaris*)		*Elymus pungens*, *E*. *repens*, *Aegilops triuncialis*, *D*. *glomerata*, *B*. *phoenicoides*	*E*. *pungens*, *E*. *repens*, *A*. *triuncialis*, *D*. *glomerata*
	Lasieso	*B. garciae*		*D*. *glomerata*, *Helictotrichon bromoides*, *E*. *repens*	*D*. *glomerata*, *H*. *bromoides*, *E*. *repens*
	Caldearenas	*B. garciae*		*E*. *repens*, *D*. *glomerata*, *Aegilops ventricosa*	*E*. *repens*, *D*. *glomerata*
Albarracín, Teruel	Torres de Albarracín	*B. garciae*	Wheat (14%), barley (20%), triticale (61%), rye (1%), oats (3%)	*D. glomerata*	*D. glomerata*
	Bronchales	*B. garciae*		*D*. *glomerata*, *A*. *ventricosa*, *Festuca* sp.	*D*. *glomerata*, *A*. *ventricosa*
Gúdar, Teruel	Cedrillas	*B. garciae*	Wheat (63%), barley (24%), triticale (9%), rye (1%), oats (3%)	*Hordeum murinum*, *A*. *ventricosa*, *E*. *repens*, *D*. *glomerata*	*H*. *murinum*, *A*. *ventricosa*, *E*. *repens*, *D*. *glomerata*
	Monteagudo del Castillo	*B. garciae*	Wheat (59%), barley (26%), triticale (14%), oats (1%)	*H*. *bromoides*, *Aegilops ovata*, *H*. *murinum*, *D*. *glomerata*	*H. bromoides*
	Allepuz	*B. garciae*	Wheat (36%), barley (24%), triticale (25%), oats (15%)	*B*. *phoenicoides*, *H*. *bromoides*, *A*. *ovata*, *H*. *murinum*, *D*. *glomerata*, *Bromus* sp., *Phleum phleoides*	
Campo de Montiel, Albacete	El Ballestero	*B. hispanica*	Wheat (35%), barley (53%), oats (12%)	*D*. *glomerata*, *A*. *ovata*, *Festuca* sp., *Avena fatua*, *E*. *repens*	*D. glomerata*

^a^ Percentage was estimated from data provided by Sección de estadística del Departamento de Agricultura, Ganadería y Medio Ambiente del Gobierno de Aragón and MAGRAMA (2020).

Huesca and Teruel provinces are located in the Ebro River basin area in north‐east Spain (Figure 1). The Ebro River basin has a typical Mediterranean continental climate with an annual average temperature of 9 to 11°C, cold winters below 0°C and hot summers exceeding 30°C during June to September. Annual average rainfall in this region ranges from 300 mm in the central basin (Lleida) to more than 1000 mm in the mountainous extremes of the Pyrenees in the north (Huesca) and Iberian System in the south (Teruel). Winter and summer are the driest periods and over 70% of the rainfall occurs in spring and autumn. Albacete province is in south‐eastern Spain at an altitude of 1000 m a.s.l. and has a mild Mediterranean continental climate with average temperature of 14.2°C with short cold winters and summer days exceeding 35°C (Table S2). Annual average rainfall in this area is much lower than that of the northern central Pyrenees and Iberian System range.

During surveys, the GPS coordinates at each location were recorded with a Garmin GPSmap 60CSx instrument. The survey data included a detailed description of morphological features and phenology of the barberry, presence or absence of aecia, level of aecial infection if present, presence of rust on nearby cereal crops and grasses, and infection level if present. The severity of aecial infection on barberry was visually estimated as an averaged percentage of the infected surface on the leaves of randomly selected bushes from which samples were taken. Sampled plants of *Berberis* spp. during the survey were later keyed to species based on morphological parameters, as described in Flora Iberica (Lopez González, 1986). Sampled grasses were initially keyed to respective species by the authors following classifications by Romero (2011). Identifications were later confirmed or corrected by specialists at the University of Lleida. At least one barberry sample from each site was prepared as a herbarium specimen for future reference. Samples of infected barberry leaves were collected in glassine bags and air dried at room temperature for 48 h. Sample bags were identified with a unique sample code, name of plant species, site and date of collection. Dried infected barberry samples were mailed to the Biosafety Level 3 (BSL‐3) Plant Pathogen Containment Facility at the USDA‐ARS Foreign Disease‐Weed Science Research Unit at Ft Detrick (MD, USA). Shipping and receiving protocols followed USDA APHIS PPQ permit conditions for handling international samples of *P*. *graminis*.

### 2.2 Inoculation assay on identification series

A set of genotypes including wheat (*Triticum aestivum* ‘Morocco’ and Line E), barley (*Hordeum vulgaris* ‘Hiproly’), rye (*Secale cereale* ‘Prolific’) and oat (*Avena sativa* ‘Marvelous’) was used for initial inoculation experiments. These cultivars are known to be “universal” susceptible genotypes of the cereal crop species that serve as the aeciospore infection hosts to provide a preliminary identification of Pgt, f. sp. *secalis* (Pgs, rye stem rust pathogen) and f. sp. *avenae* (Pga, oat stem rust pathogen). Bulked barberry leaf samples bearing aecia were used to inoculate seedling plants of the above genotypes following the procedure of Jin et al. (2010). This inoculation method allows the release of aeciospores from aecia placed above the seedlings in a moisture‐saturated environment in a dew chamber. When aecial samples were small, aeciospores were collected into gelatin capsules, suspended in mineral oil and sprayed onto the seedling plants following a procedure that was similar to urediniospore inoculation (Jin et al., 2007). An infection period of 48 h was provided in both inoculation methods to facilitate adequate spore rehydration, germination and infection to take place. After the infection period, plants were moved to a growth chamber cycling between 20°C/14‐h light and 18°C/10‐h darkness for further incubation. Infections were recorded 14 days postinoculation (dpi) and resultant urediniospores were collected for further analysis.

### 2.3 DNA extraction and ITS sequencing of aecial samples

Genomic DNA was extracted from single aecial pustules using the OmniPrep DNA extraction kit (G‐Biosciences), following the manufacture's protocol for fungal tissues. A segment of approximately 1300 bp containing the 5′ end of the 18S rRNA, complete internal transcribed spacer (ITS) region and 5′ end of the 28S rDNA was amplified using the primer pair ITS1‐F (Gardes & Bruns, 1993) and RUST1 (Kropp et al., 1995). PCRs were conducted in total reaction volumes of 50 μl (0.25 mM of each primer, 100 µM of each dNTP, 0.75 U *Taq* DNA polymerase, 10× standard *Taq* buffer [NEB N0273] and 50–100 ng of template DNA). Cycling conditions consisted of 5 min at 94°C; 32 cycles of 30 s at 94°C, 30 s at the appropriate primer annealing temperature and 15 s at 68°C; followed by a final 5 min elongation step. PCR products were purified using Wizard SV Gel and PCR Clean‐Up System (Promega), and the purified products were cloned into the pCR4‐TOPO TA vector (ThermoFisher Scientific) according to the manufacturer's instructions. Prior to sequencing, the cloned plasmids were purified using QIAprep Spin Miniprep Kit (QIAGEN). The inserts of eight recombinant plasmids per amplicon were then bidirectionally sequenced with the primers M13F and ITS4BM using Sanger sequencing at the Genomic Center, University of Minnesota, USA. A similar approach was used to clone and sequence the corresponding region from *Cumminsiella mirabilissina* (HSZ1967).

Raw sequences were assembled using the de novo assemble function in Geneious Prime (http://www.geneious.com). After trimming the partial 18S and 28S rRNA regions, sequences from the eight recombinant plasmids were multi‐aligned and consensus sequences for each sample were generated. Final error‐corrected consensus sequences were deposited in GenBank (accession numbers in Table S3).

### 2.4 Identification of rust pathogens based on ITS sequence

Initial identifications of *Puccinia* spp. from aecial samples were made by comparing the complete ITS1, 5.8S rRNA and ITS2 regions against all GenBank entries using BLASTN (http://blast.ncbi.nlm.nih.gov/). A subsequent phylogenetic analysis was performed to confirm the BLAST results. Because barberry can host multiple species of *Puccinia*, reference ITS sequences of 20 *Puccinia* spp. (Barnes & Szabo, 2007; Berlin et al., 2013) and one *C*. *mirabilissina* (authors’ unpublished data), all of which can infect barberry, were obtained from GenBank. Sequence alignment of the ITS regions from the aecial samples and reference ITS sequences were generated. Phylogenetic analysis was performed in Geneious Prime (https://www.geneious.com) using a neighbour‐joining approach with bootstrap values determined from 5000 replicates. The sequence of *C*. *mirabilissina* was used as an outgroup.

## 3 RESULTS

### 3.1 Barberry species, their distribution and cereal production system

**FIGURE 2:**
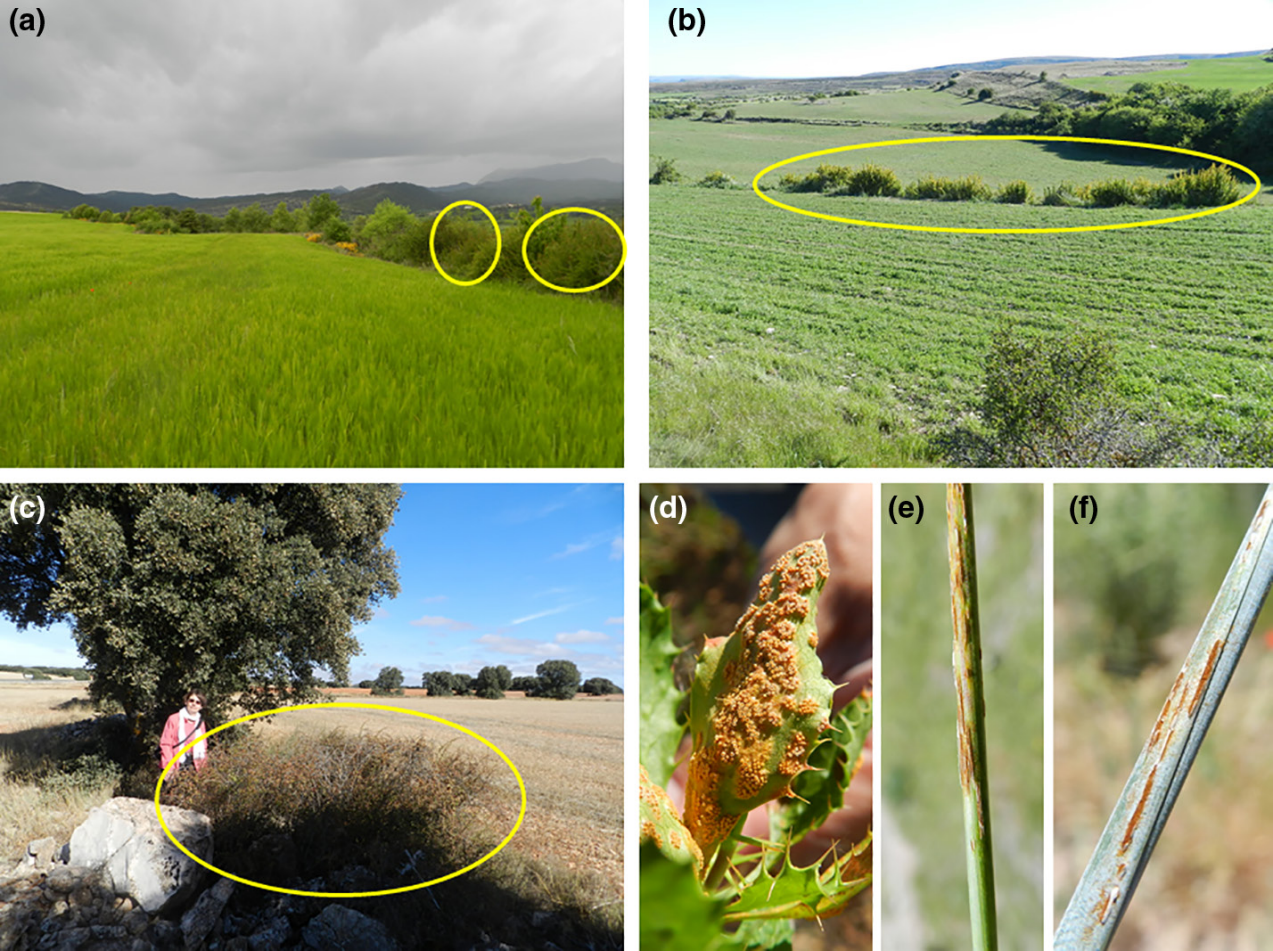
Barberry bushes (circled) in field margins in (a) Huesca province, (b) Teruel province and (c) Albacete province; (d) aecial infection on *Berberis garciae* in Teruel province in May 2019; (e) stem rust uredinia on *Elymus repens* in Teruel province in June 2019; (f) stem rust infection on triticale in Teruel province in June 2019

*B*. *garciae* was the predominant species in Huesca and Teruel provinces (Figure 1). Some rare hybrids of *B*. *garciae* and *B*. *vulgaris* were also identified at Hostal de Ipiés in the Jaca area (Table 1). In both provinces, *B*. *garciae* was found at altitudes from 600 to 1500 m a.s.l. where cereal crops are widely grown. Barberry bushes were often found at the field margins and in very close proximity (0–25 m) to cereal crops. Various grasses were common in the field margins together with barberry bushes (Figure 2). In the Jaca area of Huesca province (Figure 2a), barberry was present as isolated plants or in clusters at the field margins of fewer than 15% of fields. In Teruel province, especially the Gúdar area, barberry was very common and found in the margins of more than 50% of the fields, often forming large clusters. In this area, barberry was frequently in close proximity to cereal fields (Figure 2b). At the Campo de Montiel site in Albacete province in the south (Figure 2c), *B*. *hispanica* was found at higher altitudes, approximately 1000 m a.s.l. Although barberry bushes at this site were scattered and found in less than 5% of the area, they were close to cereals crops (Figure 2c). *B*. *hispanica* can be found up to 2500 m a.s.l. (López González, 1986), but cereal crop cultivation rarely occurs at that altitude.

The most widely grown cereal crops at the surveyed sites were wheat, barley and triticale, with occasional oats and rye. The proportions of each crop differed between sites (Table 1). Barley and wheat were predominant followed by triticale in the Jaca area in Huesca province (Table 1). Triticale was the most widely grown crop in the Albarracín area in Teruel province, followed by barley and wheat (Table 1). In the Gúdar area in Teruel province, wheat was predominant, followed by barley and triticale. Barley was the most important crop in Albacete province, followed by wheat and oats (Table 1).

### 3.2 Phenology of rust on barberry, grasses and crops

**FIGURE 3:**
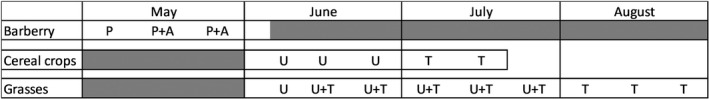
Stages of rust development on aecial and telial hosts in Huesca province. Grey shading, plants free of rust infection. Letters in text box represent the stage of rust presence. P, pycnia; A, aecia; U, uredinia; T, telia

At the onset of spring, *B*. *garciae* in Huesca and Teruel provinces “leafed out” in April and flowered in May. Aecial infections on barberry (Figure 2d) appeared in May, coinciding with the flowering period of barberry. Aecial infections were most frequently observed on barberry leaves and occasionally on flowers and immature fruits. The presence of active aecia on barberry lasted less than one month, after which lesions became necrotic. New growth of barberry leaves without rust infection followed (Figure 3). At Campo de Montiel in Albacete province there were active aecial infections on *B*. *hispanica* in June when cereal crops were near maturity or already harvested. Most of the barberry bushes surveyed in this area were healthy, with only a few having aecial infections.

In Jaca sites (Huesca province), infection in cereal crops was not observed in 2018 nor in previous years. However, stem rust infections on several cereal crop species were observed and sampled in 2019, but the impact of the disease on yield was minimal in most cases (personal communication with farmers). Infections in crops began during the first half of June, and severity was highest in later ripening crops. In early June, infections on barley were observed at low severity, when close to grain maturity (DC 80–85, Zadoks et al., 1974) and growing 0–12 m from barberry bushes. Late wheat varieties showing initial infections were at anthesis at that time and disease levels increased during late June. An oat field at the grain‐filling stage located 70 m from barberry bushes had light stem rust infection in mid‐June. By early July, only the late wheat and rye varieties at the dough stage (DC 85) remained green and these carried stem rust uredinia, whereas all other cereal crops were already mature, but with stem rust telia in severities lower than 30% only when close (less than 15 m) to barberry.

Stem rust uredinia were observed at the Teruel survey sites in cereal crops in early to mid‐June in both survey years (2018 and 2019; Figure 2f). The first symptoms of stem rust in wheat were observed by the end of June in wheat at the milk stage of grain development (DC 75). At the same time, rye was at anthesis and had initial stem rust symptoms. In the Gúdar area (Figure 1), stem rust infections in rye were very severe in 2018 and totally infected rye crops still remained in September, whereas normal harvest is in July or August. Most of the fields that had infections in 2018 were in fallow or were grown with non‐cereal crops (sunflower or vetch) in 2019, the usual practice for weed control in the area.

Aecia were observed on barberry in Albacete province during the first week of June when the majority of wheat crops were already mature or close to maturity. Infections on crops or weeds were not detected in 2019, but telia were observed in *Dactylis glomerata* in 2018. Lowland cereal crops (200–300 m a.s.l.) in the south of Spain are usually harvested by the end of May or beginning of June.

Grass species that could potentially serve as accessory hosts for cereal stem rust pathogens and those with stem rust infections are given in Table 1. When present, *Elymus repens* and *Elymus* *pungens* were usually infected with stem rust (Figure 2e), and both species showed late and staggered heading times with established uredinial infections at all surveyed sites, except those in Albacete province. *Aegilops triuncialis* in the Jaca area and *Aegilops* *ventricosa* at Albarrancin sites carried stem rust infections. Although *Aegilops* *ovata* and *Hordeum murinum* were found at the Allepuz site of Teruel province, there was no stem rust on these grasses. *D*. *glomerata*, one of the most common grasses across sites, was infected with stem rust at all surveyed sites. *Helictotrichon bromoides* was found occasionally with stem rust infection. *Brachypodium phoenicoides* was common at some sites, but very rarely found with stem rust infection. Stem rust‐infected *Avena* species (*A*. *sterilis* and *A*. *fatua*) were found in fields or field margins at a very low frequency. These were not included in Table 1 because they were not common.

### 3.3 Infection results on identification series hosts

Aeciospore viability was lost during storage and shipment for all aecial samples received at USDA‐ARS Cereal Disease Laboratory (CDL) in 2018, thus no data were obtained. Samples received from 2019 were relatively fresh; 12 of the 29 aecial samples produced successful infections on the identification (ID) series (summarized in Table 2). Stem rust infections occurred on wheat, barley, rye and oat genotypes, indicating the presence of Pgt, Pgs and Pga. The composition of *formae speciales* varied between samples and regions. Isolates derived from these aecial samples are currently being analysed at the CDL.

**TABLE 2:** Production of uredinia on cereal crop species and genotypes inoculated with aeciospores from infected barberry

Sample ID	Site (province)	Wheat		Barley	Rye	Oat
		Line E	Morocco	Hiproly	Prolific	Marvelous
19SPA0119	Hostal de Ipiés (Huesca)	−	+	+	+	+
19SPA0120	Larrés (Huesca)	+	+	+	+	−
19SPA0123	Larrés (Huesca)	−	−	+	−	−
19SPA0140	Larrés (Huesca)	+	+	+	+	−
19SPA0144	Larrés (Huesca)	−	−	+	−	−
19SPA0121	Lasieso (Huesca)	−	−	−	−	+
19SPA0122	Caldearenas (Huesca)	−	−	+	+	−
19SPA0124	Bronchales (Teruel)	+	+	+	−	−
19SPA0125	Torres de Albarracín (Teruel)	+	+	+	+	+
19SPA0126	Monteagudo (Teruel)	+	−	+	+	+
19SPA0127	Cedrillas (Teruel)	+	+	+	+	+
19SPA0128	El Ballestero (Albacete)	+	−	+	+	+

Note

Isolates infecting Morocco and Line E wheat and Hiproly barley are expected to be *Puccinia graminis* f. sp. *tritici*, infecting Line E and Prolific rye expected to be f. sp. *secalis* or their hybrids, and infecting Marvelous oat expected to be f. sp. *avenae*.

### 3.4 Identification of *Puccinia* spp. based on ITS

**FIGURE 4:**
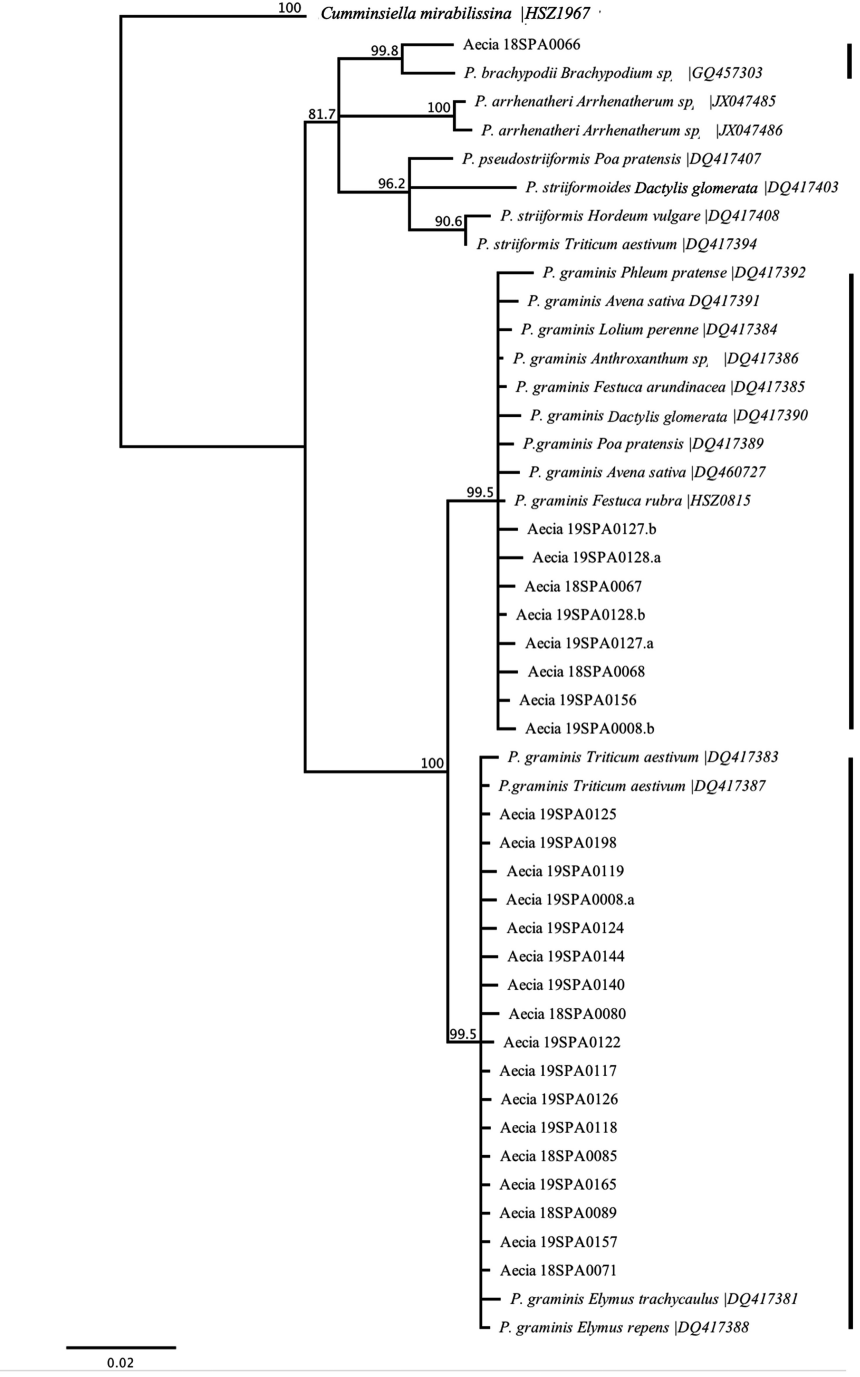
Phylogenetic analysis of the internal transcribed spacer (ITS) region from 22 aecial samples collected in Spain during 2018 and 2019. Included in the analysis are 22 reference sequences. The *Cumminsiella mirabilissina* sequence was used as an outgroup. Bootstrap values for 5000 replicates are shown (>75%)

Of the total 51 aecial samples received at CDL in 2018 and 2019, the 1.3 kb region containing partial 18S, complete ITS sequence and partial 28S rDNA was successfully amplified and sequenced for 22 samples. BLAST analysis showed 21 of 22 samples had best hits with *P*. *graminis* and shared very high similarity (E‐value: 0%, ID: 99.1%–100%; Table S3). One sample, 18SPA066, shared high similarity with *P*. *brachypodii* (E‐value: 0%, ID: 98%). Neighbour‐joining analysis of the ITS region with 21 reference isolates confirmed the identification of *P*. *graminis* in 21 of the 22 aecial samples (Figure 4). Subsequent phylogenetic analysis further grouped the aecial samples into two distinct clades; 16 samples were grouped with *P*. *graminis* from wheat (*T*. *aestivum*) and grass species *E*. *repens* and *Elymus* *trachycaulus*; five aecial samples grouped with *P*. *graminis* from oat (*A*. *sativa*) and other grasses (*D*. *glomerata*, *Lolium perenne*, *Phleum pratense*, *Festuca arundinacea*, *Festuca* *rubra*, *Poa pratensis* and *Anthroxanthum* spp.). Sequences could not distinguish between *P*. *graminis* genotypes specialized to wheat, rye and *Elymus* spp., or between *P*. *graminis* of oats and other wild grasses.

## 4 DISCUSSION

There has been growing global concern regarding the re‐emergence of wheat stem rust after many decades of quiescence. Once feared as the “bubonic plague of wheat”, this disease was deemed to be under control until race Ug99, with virulence to the widely deployed resistance gene *Sr31*, caused serious outbreaks in East Africa and made an estimated 80% of the world's wheat germplasm vulnerable to stem rust (Singh et al., 2011). In the last 20 years, multiple stem rust outbreaks and epidemics have been reported in Asia, Africa and in Europe, which had not experienced significant stem rust outbreaks in the previous 50 years (Olivera Firpo et al., 2017; Olivera et al., 2015, 2019; Saunders et al., 2019). As several countries in Europe and other parts of the world were experiencing a resurgence of stem rust in wheat, more intensive searches revealed a concurrent increase in the prevalence of common barberry (*B*. *vulgaris*) in wheat‐growing regions and provoked questions regarding the potential role of other *Berberis* species in cereal rust epidemiology. The *Berberis* genus is highly diverse, with different species distributed nearly worldwide, and centres of diversity in southern and eastern Asia as well as Central and South America (Ahrendt, 1961).

In our case, the field survey conducted in Huesca and Teruel provinces in Spain revealed widely distributed barberry populations and frequent co‐occurrence of indigenous barberry with cereal crops and grasses that potentially serve as accessory hosts of cereal stem rust pathogens. *B*. *garciae* and interspecific hybrids of *B*. *garciae* and *B*. *vulgaris* in these provinces were pervasive and often close to cereal crops. Abundant aecial infections were observed under natural conditions. More importantly, stem rust infections in cereal crops and grasses were found during May to July following aecial infection on barberry. This suggested that aecial infections on barberry probably provided the primary sources of inoculum for stem rust infections of cereal crops in Huesca and Teruel provinces, although further investigation is needed to confirm this relationship. This contrasted with the presence of *B*. *hispanica* in Albacete province where barberry was rarely found close to cereal crops, as barberry was seldom in the field margins. More significantly, active infections on barberry were found only after cereal crops were harvested or approached maturity in June. Located in south‐eastern Spain, Albacete province has a relatively warmer spring than the northern provinces and crops mature earlier. Thus, aecial infection on barberry rarely coincides with the vegetative growth period of cereal crops, and barberry is less likely to provide primary inocula to cereal crops in this region.

Inoculation experiments using aecial samples resulted in successful stem rust infection on all cereal crop species (wheat, barley, rye and oat) used in this study. DNA ITS sequence analysis identified the presence of multiple *P*. *graminis* forms. These results corroborated field observations and unequivocally proved that *Berberis* spp. are functional as the alternate host for *P*. *graminis* in this region. Although the ITS sequence analysis cannot distinguish between *P*. *graminis* f. sp. *tritici* and f. sp. *secalis*, the results of inoculation experiments indicated the presence of both forms and possible sexual hybrids between both groups. The presence of *P*. *graminis* f. sp. *avenae* was also confirmed, evidenced by infection of cv. Marvelous oat. It is also important to note that many wild grasses such *D*. *glomerata*, *Elymus* spp., *A*. *triuncialis*, *A*. *ventricosa*, *H*. *murinum* and *H*. *bromoides* were infected with stem rust. Because each grass species has a distinct phenology, grasses in general may carry stem rust uredinia for longer time periods than cereal crops. For example, *Elymus* spp. can harbour uredinia for extended periods as they remain green during most of the summer. Because most wild grasses remain undisturbed by human activities at the field margins and uncropped areas, they probably sustain *P*. *graminis* when crops are absent and produce telia for overwintering to serve as a source of inoculum to infect barberry bushes the following spring. In this regard, the wild grasses that can serve as the accessory hosts of *P*. *graminis* have probably played an important role in the epidemiology of stem rust in this region. However, the question remains as to which, if any, of the grass species are potential uredinial hosts for cereal‐infecting forms. Identification of the host virulence aspects of uredinial samples from grasses will be a necessary adjunct to field surveys of infections on both cereals and potential alternate hosts. The fact that fallow and non‐cereal crop rotations observed in Teruel did not prevent stem rust infection in cereals in this region reinforces the idea that grasses may have played an important role in sustaining infections without the presence of cereal crops. If this role could be confirmed, management of weedy grasses near barberry plants along the field margins would provide a potential novel approach for stem rust control.

It is well established that the sexual cycle of the stem rust pathogen on barberry allows the development of new races following genetic recombination among loci (Jin, 2011; Roelfs, 1982). However, there must be a coincidence of aeciospore release and the presence of a receptive telial host. Earlier maturing autumn‐sown cereals in some regions were a major contributor in reducing the risk of stem rust. The ubiquitous co‐occurrence of susceptible barberry adjacent to cereal crops and accessory grass hosts observed in this study is concerning. Although pathogen strains with novel virulence or unique virulence combinations generated through sexual cycles may not be favoured by selection in the short term, avirulence/virulence polymorphisms will continuously evolve and be sustained in the local population. This may incite epidemics many years later, such as the epidemics of race 15B in the United States (Jin, 2011).

The widespread occurrence of barberry adjacent to cereal crops is equally concerning for stripe (yellow) rust pathogens of wheat, triticale and barley. Although *P*. *striiformis* is not currently known to undergo sexual reproduction in Europe (Rodríguez‐Algaba et al., 2021) and *P*. *striiformis* was not detected from aecial samples in this study, there has been increasing concern that the sexual stage of *P*. *striiformis* is already established in Europe (Lewis et al., 2018). Recently Rodríguez‐Algaba et al. (2021) found that the same *Berberis* spp. are susceptible to the wheat stripe rust pathogen in controlled inoculation experiments. The ubiquitous existence of indigenous and susceptible barberry alongside cereal crops presents an opportunity for the sexual cycle to take place in the region if conducive environmental conditions are met.

This study provides the first evidence that indigenous *Berberis* spp. play an active role in the sexual cycle of *P*. *graminis* under natural conditions in Spain. Further investigations are needed to ascertain the specific roles of alternate hosts (*Berberis* spp.) and accessory hosts in cereal rust epidemiology in different regions of Spain so that appropriate control strategies can be developed. The role of the alternate host in generating pathogen variation is currently being investigated through uredinial race analysis of isolates derived from cereals and grasses and from aecial samples collected on *Berberis* spp.

## Supporting information

Table S1

Table S2

Table S3

## Data Availability

The ITS sequence data of aecial samples report in this this article are deposited in GenBank under the accession numbers listed in Table S3. Herbarium specimens for *Berberis* spp. were deposited at the Herbarium of the Pyrenean Institute of Ecology (IPE‐CSIC) in Jaca (Spain) under the herbarium deposit reference numbers JACA R310195 to JACA R310207.
