# Supplementary material for: Barberry plays an active role as an alternate host of Puccinia graminis in Spain
Source: Plant Pathol. 2022 Mar 9;71(5):1174–84. doi: 10.1111/ppa.13540 (PMC9311844; doi:10.1111/ppa.13540)
Supplement: Supplementary file 1 — Table S1 [file PPA-71-1174-s002.docx]

Supplementary **TABLE S1**. Sampling calendar and the rust life stage observed in 2019.

| **Date** | **Province** | **Locations** | **Rust life stage of sample** |
| --- | --- | --- | --- |
| May 1 | Huesca | Lasieso, Hostal de Ipiés | Initial infection on barberry, telia on remains of grasses |
| May 11 | Huesca | Larrés, Lasieso, Hostal de Ipiés | Pycnia on barberry |
| May 18 | Huesca | Larrés, Lasieso, Hostal de Ipiés, Caldearenas | Actively sporulating aecia on barberry |
| June 1 | Teruel | Bronchales, Torres de Albarracín, Monteagudo del Castillo, Cedrillas | Active aecia on barberry |
| June 1 | Albacete | El Ballestero | Active aecial infection on barberry, with incipient necrosis (end of infection) |
| June 16 | Huesca | Larrés, Hostal de Ipiés, Lasieso, Caldearenas | Low severity aecial infections on barberry. Profuse infection on cereal crops and grasses |
| June 22 | Teruel | Bronchales, Torres de Albarracín, Allepuz, Monteagudo del Castillo, Cedrillas | Active aecia on barberry, uredinia on cereal crops and grasses |
| July 7 | Huesca | Larrés, Hostal de Ipiés, Lasieso, Caldearenas | Stem rust infection on grasses, with mixture of uredinia and telia |
| August 24 | Huesca | Larrés, Hostal de Ipiés, Lasieso | Sporadic uredinia on grasses |
