# Supplementary material for: Barberry plays an active role as an alternate host of Puccinia graminis in Spain
Source: Plant Pathol. 2022 Mar 9;71(5):1174–84. doi: 10.1111/ppa.13540 (PMC9311844; doi:10.1111/ppa.13540)
Supplement: Supplementary file 2 — Table S2 [file PPA-71-1174-s003.docx]

Supplementary **TABLE S2**. Location and main environmental attributes of surveyed sites

| **Province** | **Location** | **Site** | **Latitude** | **Longitude** | **Elevation** | **T(ºC)** | **P (mm)** | **Eto (mm)** |
| --- | --- | --- | --- | --- | --- | --- | --- | --- |
| Huesca | Jaca | Larrés | 42.56 N | 0.39 W | 870 | 9.5 | 925 | 825 |
|  |  | Hostal de Ipies | 42.44 N | 0.39 W | 715 |  |  |  |
|  |  | Lasieso | 42.42 N | 0.44 W | 729 |  |  |  |
|  |  | Caldearenas | 42.40 N | 0.50 W | 641 |  |  |  |
| Teruel | Albarracín | Torres de Albarracín | 40.40 N | 1.50 W | 1189 | 11.1 | 463 | 838 |
|  |  | Bronchales | 40.52 N | 1.58 W | 1486 |  |  |  |
|  | Gúdar | Cedrillas | 40.44 N | 0.86 W | 1355 | 10.2 | 490 | 817 |
|  |  | Monteagudo del Castillo | 40.46 N | 0.81 W | 1442 |  |  |  |
|  |  | Allepuz | 40.49 N | 0.76 W | 1376 |  |  |  |
| Albacete | Campo de Montiel | El Ballestero | 38.81 N | 2.45 W | 983 | 14.2 | 397 | 1200 |

T: Annual average temperature; P: Annual precipitation; ET_0_: Annual reference evapotranspiration
