# Supplementary material for: Barberry plays an active role as an alternate host of Puccinia graminis in Spain
Source: Plant Pathol. 2022 Mar 9;71(5):1174–84. doi: 10.1111/ppa.13540 (PMC9311844; doi:10.1111/ppa.13540)
Supplement: Supplementary file 3 — Table S3 [file PPA-71-1174-s001.docx]

Supplementary **TABLE S3**. Best BLASTN hit results for ITS sequences of 22 aecial samples collected during 2018 and 2019 rust survey in Spain

| **Aecial sample** | |  | | **BLASTN results** | | | | | | | |
| --- | --- | --- | --- | --- | --- | --- | --- | --- | --- | --- | --- |
| **Sample ID** | **GenBank accession No.**^1^ |  | | ***Puccinia* spp.** | | **Host** | **% identity** | **Total length** | **Mismatch** | **Gaps** | **GenBank accession No.**^2^ |
| 18SPA0066 | OM265439 |  | *P. brachypodii* | | *Brachypodium* sp. | | 98.0 | 971 | 10 | 4 | GQ457303.1 |
| 18SPA0067 | OM265440 |  | *P. graminis* | | *Lolium perenne* | | 99.9 | 969 | 0 | 1 | DQ417384.1 |
| 18SPA0068 | OM265441 |  | *P. graminis* | | *Avena sativa* | | 99.9 | 970 | 0 | 1 | DQ460727.1 |
| 18SPA0071 | OM265442 |  | *P. graminis* | | *Triticum aestivum* | | 99.6 | 975 | 0 | 1 | DQ417382.1 |
| 18SPA0080 | OM265443 |  | *P. graminis* | | *Triticum aestivum* | | 99.4 | 969 | 2 | 3 | DQ417378.1 |
| 18SPA0085 | OM265444 |  | *P. graminis* | | *Triticum aestivum* | | 99.9 | 972 | 0 | 1 | DQ417382.1 |
| 18SPA0089 | OM265445 |  | *P. graminis* | | *Triticum aestivum* | | 99.9 | 972 | 0 | 1 | DQ417382.1 |
| 19SPA0117 | OM265446 |  | *P. graminis* | | *Triticum aestivum* | | 99.9 | 972 | 0 | 1 | DQ417382.1 |
| 19SPA0118 | OM265447 |  | *P. graminis* | | *Triticum aestivum* | | 99.8 | 972 | 0 | 2 | DQ417382.1 |
| 19SPA0119 | OM265448 |  | *P. graminis* | | *Triticum aestivum* | | 99.8 | 973 | 0 | 2 | DQ417382.1 |
| 19SPA0122 | OM265449 |  | *P. graminis* | | *Triticum aestivum* | | 99.8 | 973 | 0 | 1 | DQ417382.1 |
| 19SPA0124 | OM265450 |  | *P. graminis* | | *Triticum aestivum* | | 99.8 | 972 | 0 | 2 | DQ417382.1 |
| 19SPA0125 | OM265451 |  | *P. graminis* | | *Triticum aestivum* | | 100.0 | 971 | 0 | 0 | DQ417382.1 |
| 19SPA0126 | OM265452 |  | *P. graminis* | | *Triticum aestivum* | | 99.8 | 973 | 0 | 1 | DQ417382.1 |
| 19SPA0127 | OM265453 |  | *P. graminis* | | *Avena sativa* | | 99.9 | 970 | 0 | 1 | DQ460727.1 |
| 19SPA0128.a | OM265454 |  | *P. graminis* | | *Dactylus glomerata* | | 99.1 | 969 | 1 | 2 | DQ417390.1 |
| 19SPA0140 | OM265455 |  | *P. graminis* | | *Triticum aestivum* | | 99.6 | 971 | 2 | 2 | DQ417382.1 |
| 19SPA0144 | OM265456 |  | *P. graminis* | | *Triticum aestivum* | | 99.7 | 973 | 0 | 2 | DQ417382.1 |
| 19SPA0156 | OM265457 |  | *P. graminis* | | *Anthoroxanthum* sp. | | 99.7 | 969 | 2 | 1 | DQ417386.1 |
| 19SPA0157 | OM265458 |  | *P. graminis* | | *Triticum aestivum* | | 99.7 | 974 | 0 | 1 | DQ417382.1 |
| 19SPA0165 | OM265459 |  | *P. graminis* | | *Triticum aestivum* | | 99.9 | 972 | 0 | 1 | DQ417382.1 |
| 19SPA0198 | OM265460 |  | | *P. graminis* | *Triticum aestivum* | | 99.9 | 972 | 0 | 1 | DQ417382.1 |

^1^ GenBank accession number of the ITS sequence of aecial sample used in this study.

^2^ GeneBank accession number of the best BLASTN hit *Puccinia* spp. for the aecial sample used in this study.
